# Physical Activity Recommendations during COVID-19: Narrative Review

**DOI:** 10.3390/ijerph18010065

**Published:** 2020-12-24

**Authors:** Patricia Polero, Carmen Rebollo-Seco, José C. Adsuar, Jorge Pérez-Gómez, Jorge Rojo-Ramos, Fernando Manzano-Redondo, Miguel Ángel Garcia-Gordillo, Jorge Carlos-Vivas

**Affiliations:** 1 Laboratorio de Biomecánica y Análisis del Movimiento del Litoral, CENUR Litoral Norte, Universidad de la República, Florida 1065, Paysandú 60000, Uruguay; patricia.polero@gmail.com; 2 Health, Economy, Motricity and Education Research Group (HEME), Faculty of Sport Sciences, University of Extremadura, 10003 Cáceres, Spain; jadssal@unex.es (J.C.A.); jorgepg100@unex.es (J.P.-G.); jorgerr@unex.es (J.R.-R.); fmanzanoa@alumnos.unex.es (F.M.-R.); jorge.carlosvivas@gmail.com (J.C.-V.); 3 Facultad de Administración y Negocios, Universidad Autónoma de Chile, Sede Talca 3467987, Chile; miguelgarciagordillo@gmail.com

**Keywords:** coronavirus, pandemic, physical exercise, physically inactivity, health

## Abstract

Regular practice of physical activity plays a fundamental role in preventing and treating cardiovascular diseases such as obesity, hypertension, diabetes, and metabolic syndrome. During the pandemic caused by COVID-19 and the lockdown established, people have reduced considerably their mobility and motor activity, which has led to an increase in unhealthy lifestyle habits, raising the risk of suffering from diseases. This paper consists of reviewing the existing scientific literature on recommendations of physical activity during the pandemic and to establish specific guidelines according to the type of population to which the activity would be directed. A search strategy has been carried out in the different databases: Embase, PubMed, SCOPUS, SPORTDiscus, and Web of Science (WoS), including all the articles published until 14 May 2020, to find essays with recommendations on aerobic activity, muscle strengthening, flexibility-stretching, meditation-relaxation, and balance exercises. The articles found have been evaluated considering the following criteria: type of publication, proposals for physical exercise, language, and, if it appears, volume of activity, frequency, intensity, and rest. The results obtained 29 articles that discuss patterns of physical activity, although there is no common consensus on such recommendations during confinement, nor are they suitable for all people. From these results, we can conclude that physical activity is widely recommended during the confinement caused by COVID-19, mainly through the performance of aerobic, strength, flexibility, and balance exercises.

## 1. Introduction

The COVID-19 pandemic was first reported in China at the end of 2019 [1]. It is a viral disease that affects people’s health and quality of life, causing respiratory infections such as mild and moderate pneumonia. This new virus is related to severe acute respiratory syndrome (SARS) and is also known as SARS-CoV-2 [1].

The disease has affected the entire world’s population, with confirmed cases in more than 34 million people with COVID-19, causing more than 1 million deaths worldwide [2]. Spain, Italy, and Germany were the countries with the most contagion on the European continent at the start of the pandemic, although Russia and the United Kingdom now appear as the European countries with the most diagnosed cases. Outside the European continent, the United States, India, Brazil, and Chile are the countries with the highest number of contagions [3]. Specifically, currently in Spain, nearly 778,000 COVID-19 infections and more than 31,000 deaths from COVID-19 [2] have been reported [3]. However, it should be noted that this data is updated and increased day by day until a treatment can be found.

On the one hand, according to Hall et al. [4], the containment measures adopted during the pandemic could have caused a large part of the population to adopt unhealthy lifestyles, such as physical inactivity, although data are not entirely conclusive. However, on the other hand, COVID-19 provided an opportunity for many young athletes to train more than ever as it was easier to fit in training sessions during remote schooling.

Adopting unhealthy lifestyles could have negative consequences, both in dealing with the virus today and in the quality of life of sedentary people once the pandemic has passed. Some of the consequences that this sedentary situation can cause and which can be observed over time are cardiovascular diseases, such as obesity, hypertension, diabetes, and metabolic syndrome, because decreased physical activity is linked to a reduction in insulin sensitivity [5].

In this sense, Narici et al. [5] claim that inactivity during confinement can result in a reduction in maximum oxygen consumption (VO2max), oxygen absorption capacity, and cardiac volume. A reduction in VO2max and the absorption capacity of O_2_ is associated with a higher mortality rate. In addition, this decrease affects blood circulation and the oxidative function of muscles.

In this context, physical activity is constituted as a fundamental strategy to combat these unhealthy lifestyles during this or similar situations, since it contributes to maintaining an optimal state of health, both physical and mental [6]. The World Health Organization (WHO) has proposed several general recommendations for physical activity to try to combat the confinement situation. These recommendations are aimed at the general adult population and consist of performing 150 min or 75 min per week of physical activity at a moderate or vigorous intensity, respectively. It is also recommended to combine activities at different intensities [7].

Physical activity not only helps reduce body mass or prevent cardiovascular disease, but it also helps to improve immune system function and cope with viral infections. Depending on the exercise and its intensity, the immune system makes adaptations by improving its function [8]. To show adaptations in the immune system, it is important that the intensity of exercise is moderate, as with this level intensity antipathogenic activity is improved in the immune system. In addition, moderate exercise is linked to a decrease in mortality from respiratory diseases, such as COVID-19 affecting the respiratory tract [8]. Therefore, regular physical activity can help with a better response to vaccines, which is why physical exercise and being active can help cope with this pandemic [8].

The important role that physical activity plays in people’s health and quality of life seems indisputable. Besides, the practice is a fundamental strategy to combat the possible consequences of a confinement situation such as that recently caused by COVID-19. Therefore, the objective is to review the existing literature, including studies that have proposed recommendations for the practice of physical activity during the pandemic, and to establish specific guidelines according to the type of population to which the activity would be directed. Concerning this objective, the hypothesis was raised that an adequate analysis of the scientific literature will allow knowledge of what the most recommended physical activity patterns are, depending on the type of population, to maintain physical condition levels during a period of confinement.

## 2. Methods

### 2.1. Search Strategy

**Figure 1:**
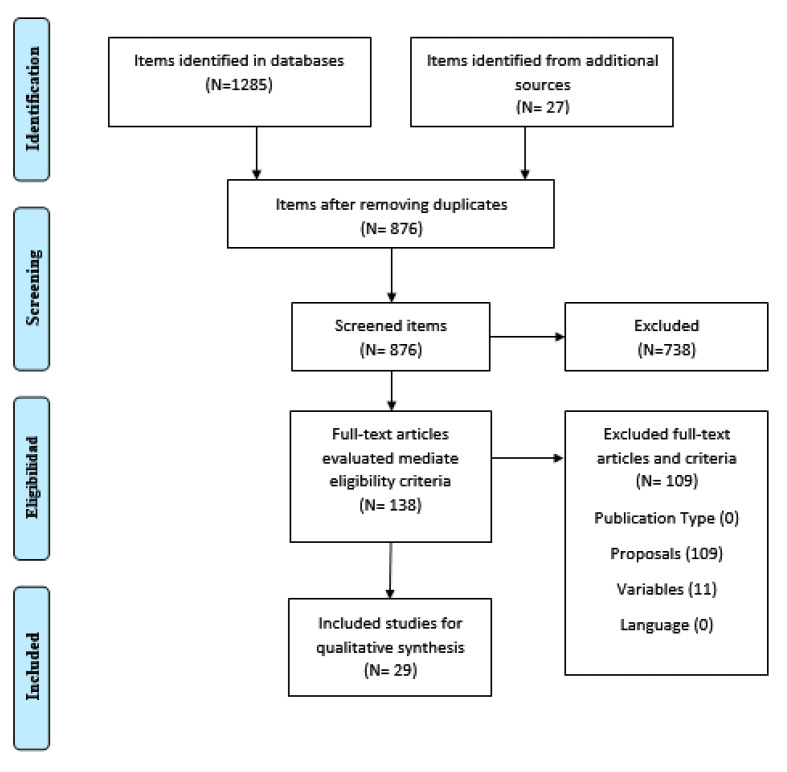
Flowchart of the process of searching and selecting the studies.

The search was carried out on five different databases: Embase, PubMed, SCOPUS, SPORTDiscus and Web of Science (WoS), including all articles published until 14 May 2020. The search strategy consisted of the combination of the following Boolean keywords and operators: (COVID-19 OR “Coronavirus 2” OR SARS-CoV-2 OR 2019-nCoV severe OR Coronavirus OR SARS OR “acute respiratory syndrome” OR “severe acute respiratory syndrome coronavirus 2” OR “Novel humancoronavirus” OR “severe acute respiratory syndrome coronavirus 2”) AND (“physical activity” OR “physical exercise” OR “physical inactivity” OR “training” OR “physically active” OR “workout program*” OR “physical fitness” OR “physical condition” OR exercise OR fitness OR “training program*”). Figure 1 shows the flowchart relating to the process of finding and selecting the studies included in this narrative review.

### 2.2. Eligibility Criteria

To be considered in this review, studies had to meet the following eligibility criteria: articles published in indexed journals; articles that make recommendations on physical activity; studies that include explanation and information on the recommendations and guidelines to follow, i.e., type of exercise, volume and intensity; and articles published in The Spanish or English language. Figure 1 shows the flowchart of the search and selection process for the included studies.

### 2.3. Removing and Analyzing Data

The data extracted has been evaluated by two researchers who independently reviewed the titles and summaries of the articles found, considering the eligibility criteria. Disagreements were resolved by discussion, or in the event of no agreement by a third reviewer or researcher.

## 3. Results

Initially, a total of 1312 studies were identified in the different databases. After eliminating duplicates, there were 876 studies, of which 738 were excluded after reading their title and abstract. Therefore, 138 articles were revisited in full text and evaluated according to the eligibility criteria, with 109 articles being deleted, by not meeting each one of the proposed criteria. Finally, this review includes a total of 29 articles. In these articles, we can find recommendations for a single population group or guidelines for more than one population.

### 3.1. Aerobic Activity Recommendations

Table 1 shows articles that include guidelines or recommendations on aerobic work. A total of 28 items include aerobic exercise [5,6,7,9,10,11,12,13,14,15,16,17,18,19,20,21,22,23,24,25,26,27,28,29,30,31,32,33,34]. The most recommended activities are walking, climbing stairs, performing activities on static machines, and running, among others. Depending on the type of population the frequency varies from once a day for the young people, and between 2–3 times per week or between 5–7 days for adults or older people. Recommend workloads range from 150 min to 300 min per week with moderate intensity (effort 5–6 on a scale of 1 to 10) or 75 min at a vigorous intensity (effort 7–8 on a scale of 1 to 10) population; while, for school- or preschool-aged children, between 60 and 180 min of activity per day is recommended.

### 3.2. Strength Activity Recommendations

Table 2 shows [5,6,7,9,10,11,13,14,15,16,17,18,24,25,26,27,28,29,30,31,32,33] articles that include guidelines or recommendations on strength training work. A total of 21 items include strength activity. From these studies, the most recommended exercises are to perform squats, abs, and push-ups, Pilates, get up and sit in the chair. Depending on the type of population, the frequency varies from 2 or 3 days per week for adults, while, for children it is recommended on 3 days per week. Working volumes range from 1–2 series, 2–4 series, or 5 series with 5 repetitions, 8–12 repetitions, 8–20 repetitions, or 10–15 repetitions. The intensity that is most recommended relates to one’s body weight. In addition, work with material such as weights or elastic bands can be done. Regarding recovery times, only two articles in the table provide this information recommending 20″–30″.

### 3.3. Flexibility-Stretching, Relaxation-Meditation Recommendations

Table 3 shows studies that include guidelines or recommendations on the work of flexibility-stretching and relaxation-meditation. A total of 15 articles present recommendations for flexibility, stretching, relaxation and/or meditation [7,9,10,12,13,16,17,18,19,20,28,29,30,31,33]. Most articles are aimed at the general population and recommend stretches whether dynamic or static and activities such as yoga. The recommended weekly frequency is given by two items, at 2–3 times a week. Workload varies by activity, usually recommended between 10–30 s for stretching. No information is given about intensity and recovery.

### 3.4. Balance Recommendations

Table 4 shows articles that include balancing recommendations. The table presents a total of seven articles [13,16,25,29,31,32,34] whose recommendations are aimed primarily at the general and older population. The most recommended activities are balance exercises, Tai Ji, and fall prevention for older peopled, along with recommendations for between 2–3 weekly sessions.

## 4. Discussion

Public health bodies have delivered an important message during the COVID-19 pandemic to adults and older people. They should be as active as possible, because inactivity can cause acute and chronic health implications, losing quality of life and becoming dependent, especially in older people [36].

Physical activity and, as a consequence, weight loss, are useful as a preventive measure to reduce COVID-19 mortality. Furthermore, regular physical activity can also improve insulin sensitivity and lower circulating insulin levels, which should lead to a public health message in order to reduce mortality [37].

Other studies, such as that of Chagas et al. [38], state that systematic physical exercise has a positive effect on the immune system and in reducing the risk of diseases, being a good way of reducing mortality. Pinho et al. [39] also point out that physically active people have significantly reduced mortality. Being physically active and reducing sedentariness during the COVID-19 pandemic is critical to reducing the risk of cardiovascular and metabolic diseases.

The present work aimed to compare and analyze studies that have proposed physical activity recommendations to combat the consequences of confinement, differentiating patterns aerobic, muscle strengthening, flexibility-stretching, meditation-relaxation, and balance exercises.

Some studies [6,16,25,32,33] have provided examples of exercises to work on strength during confinement. However, these do not distinguish between and are not suitable for all people and we would like to stress the importance of establishing patterns of physical activity during confinement which can be adapted to the whole population according to their space at home or whether they have a disease or not.

### 4.1. Aerobic Physical Activity Recommendations

Recommendations for physical activity were presented especially for adults in some studies [6,7,20,24,27], and in others for the general population as a whole [5,10,11,12,15,16,18,23,30,31]. The most recommended type of activity are walking, running, exercises on static machines, performing household chores, and going up and downstairs. Aerobic exercise at home can be facilitated by exercise specific equipment or walking through home or garden if possible [36]. Aerobic activities are recommended, as benefits are obtained both physiologically and psychologically (decreased risk of ischemic heart disease, high blood pressure, and brain-vascular accidents, increased insulin sensitivity, increased muscle mass, improved immune response and fatigue-free effort, decreased stress, improved physical perception and sleep quality, decreased risk of diabetes, metabolic syndrome, and high blood pressure; improved ability to transport oxygen; toning muscles; improved blood circulation, increased red blood cells, increased muscle strength and bone density) [40,41]. Even so, some recommendations for physical activity such as these cannot be carried out in every home, nor does everyone have access to a particular staircase or garden. For example, in Spain, there are 23,500 homes in which there are not even 10 square meters of space per inhabitant. In these homes, it is difficult to carry out this type of exercise and recommendations proposed [42]. Thereby, we suggest that, when different countries or states apply their restrictions, they also provide new recommendations based on these circumstances. In this way, a correct public health message would be created for the population, since to the best of our knowledge this information has not been presented.

The most recommended weekly frequency for the general and adult population is one session per day [12,13,31], although 3–5 or 2–3 sessions per week [10,23] are also recommended. Articles recommending one session per day refer to30–60 min session. It is important to work with these daily training volumes for 30 min and, with a frequency of 5 days a week, we can obtain the aforementioned improvements at the physiological and psychological level [41]. The volume of work per week that predominates for the general population is 150 to 300 min [10,15,18,29,30,31] whereas for the adult populations it is 150 min or 75 min, being moderate or vigorous-intensity respectively [6,7,20,24,27] These minimums have been recommended as they are necessary to obtain physiological benefits associated with a decreased risk of premature death, and a reduction in the risk of coronary heart disease, diabetes, embolism, depression, and hypertension [40,43]. Chodzko-Zajko, in his analysis of the different recommendations of physical activity for the population, showed that, with the same volumes, it is important to carry out aerobic activity in blocks of 10 min to obtain greater profits [44].

Finally, the intensity most recommended is moderate to vigorous, with a greater importance given to moderate intensity. Some articles provide concrete values. Active adults should exercise at an intensity: 50–80% Fcmax (70–90% Fcmax (moderate), 85–100% Fcmax (high), while sedentary adults have other intensities: 50–75% Fcmax (Low), 70–85% FcMax (moderate), 85–95% Fcmax (high) [6], moderate zone perceived stress scale (RPE) (zone 3/10 or 4/10) [15], 60–80% of maximum capacity [23]. Estévez-López et al. [43] by reviewing the recommendations for healthy adults showed in his study that the heart and reserve frequencies to be worked at are 55%–65% to 90% or 40%–50% to 85%, respectively [43]. In addition, the study states that vigorous activity decreases the risk of colon cancer and related digestive system [43]. Moderate intensity is recommended in this situation, as it improves immune function, reducing the incidence, duration and severity of respiratory tract infections [8].

Within this adult population, we have found recommendations aimed at athletes and adults with disease. Articles with specific recommendations for athletes [17,26], recommend activities such as swimming, cycling, or running [17] on a static machine or circuit [26]. Frequency should be 3–4 sessions per week with a duration of 60 min if it is a daily session or 30 min if two sessions are performed, and the intensity should be 75%–80% Fcmax [17]. This recommendation for duration and intensity is adequate, as it would increase the heart chamber, strengthen and increase the thickness of the heart, decrease heart rate, and increase lung capacity [45]. Rahmati-Ahmadabad & Hosseini state that high-intensity exercises, or HIIT, do not show an alteration in the immune system, so moderate intensities close to 70% FCmax are recommended [46]. According to Kananis [47], performing vigorous-intensity exercise on a regular basis may make one more susceptible to diseases of the airways, which is why it should not be recommended during COVID-19. This happens because high intensities suppress immune function. The guidelines shown to patients with different diseases and severities should contain different recommendations for physical activity, since with these activities and volumes of exercise users can obtain physiological and psychological benefits and improve their quality of life of the healthy adult population. Activities include walking, running, or exercising on static machines [9,22,33]. Recommended frequency is also similar, 5–7 days per week at a volume of 150 min per week or 30 min per session. Ahmed [9] stated that intensity should be 40%–59% Vo2max. Recommendations were also presented for over-65s [6,16,19,25,32,33]. It is important to stress that older people who were regularly and constantly active before the pandemic should aim to maintain or increase their levels of physical activity to achieve recommended levels where possible [36]. The main activities proposed consisted of walking, climbing stairs, multi-component exercise, or household chores. According to Villanueva & Fernández [48] and their prescription for physical activity in older people, these aerobic activities may be recommended as they help increase VO2max and reduce the risk of cardiovascular disease and mortality. The volumes of intensity most recommended in the population greater than 65 are 150 min or 75 min per week at moderate and vigorous intensity, respectively [19,32] or 200 to 400 min of physical activity per week with moderate intensity: 40%–60% Reserve Fc or 65–75% Fc max [25]. These guidelines provide health benefits in older people by decreasing the risk of Alzheimer’s disease, Parkinson’s, and brain-vascular diseases [14]. In addition, Villanueva & Fernández y Jiménez Pavón et al. [25,48] suggest exercising 5–7 days a week at moderate intensity (40–60% reserve FC), since in this way the effects noted previously are achieved [25,48], but above 70%FCmáx one should perform training sessions on only 3–5 days per week to avoid musculoskeletal or cardiac injuries.

For children it is recommended to become active in running, jumping, etc. The exercise volumes recommended were 60 min/day for those over 5 years old [6,7,14,29,35] and 180 min per day for younger children [7,35] achieving a total of 300 min per week [19,20]. These recommendations for aerobic activity help to improve neural growth, cognitive performance, differentiation, and survival of neurons, and in addition help to stimulate cellular and molecular components of the brain, improving responsibility, among others [40,44]. The recommended intensity in children is moderate to vigorous [6,7,14,19,29,35] as we have discussed above, as the immune system presents an adaptive response to moderate-intensity exercise [8,46].

### 4.2. Strength Training Recommendations

As for this type of work, activities have been recommended in adults [6,7,27] in the population generally or without specifying which population [5,10,11,12,13,15,16,18,24,28,29,30,31,33] are: push-ups, squats, plates, and abs; other activities that recommended are: sit and get up from a chair [10,13,28], burpees, climber, plates, hip extension, buttock bridge, and triceps bottoms [6,7,11,15,24]. These activities have been recommended because they produce an improvement in the prevention of diabetes and colon cancer, improving muscle strength and bone density [49]. The most recommended frequency was 2 sessions per week [10,18,27,29], although strength could also be performed 3 sessions per week [15]. This recommendation may be adequate, since greater benefits are obtained in muscle and cardiorespiratory state, which improves bone and functional health, reducing the risk of fall and injury’s. For both frequencies, exercise should be carried out on alternate days to avoid musculoskeletal injuries and ensure muscle rest [7,49].

For both the adult and general population, performing at least one series of 8–12 repetitions, or a stricter program of 2–3 series, either aerobic exercises, strength training or sets of combined exercises, can help improve strength, potency and bone density, prevent falls, improve balance control and prevent sarcopenia [50]. The training volume that was proposed for both populations varies: recommended are 5 series of 10–15 repetitions [7,24] or 20–30 depending on the exercise, with a recovery of 30–60 s [7], while Hammami et al. [6] recommend for beginners 1–2 series of between 5–15 repetitions depending on the exercise, and for advanced exercise 2–3 series with 20 repetitions, all this by means of an exercise program on a bike or rowing ergometer, bodyweight training, dance and active video gaming. Another option is to perform between 1–4 series of 8–20 repetitions [15].

Intensity does not vary between recommendations for the adult population and general recommendations, as all studies recommend strengthening, using bodyweight or material such as elastic bands or light weights [12,15,16,18,28,30,33]. Narici et al. [5] recommend exercising with a load of approximately 50% 1RM or, in the case of performing many repetitions, with a weight close to 30% 1RM [50]. These recommendations with intermediate loads help to improve strength level [50].

However, even though several studies [9,33] provide recommendations on performing exercises on machines or calisthenics, it is well known that, depending on the patient’s disease, physical activity patterns will be different. Therefore, we observe in all the literature reviewed that there are many general recommendations, but they do not clearly distinguish what kind of people and diseases they are aimed at. The frequency of the training that was proposed was 2–3 days/week and the training volume 2–4 series with 8–12 repetitions [9]. Vargas [51] in his study showed that muscle strengthening can be performed in people with respiratory diseases as, like healthy people, they would develop strength, power, and endurance and have no serious symptoms. A weekly frequency of 2–3 days with duration of 30 min was recommended; the volume set is 3 sets of 8–10 repetitions to achieve the above objectives. They propose light weights (250 g–500 g), using body weight, or elastic bands [9,33,51], and weight gain should be gradual at 40–50% 1RM, then 60–70% 1RM [9]. According to Vargas [51], between 50% and 80% of 1RM is suitable for people with respiratory diseases and would achieve a reduction in dyspnea and an increase in exercise tolerance, so working recommendations between 40% of 1RM and ending with 70% of 1 MRI may be suitable for this population. The over 65s should also work on muscle strengthening during confinement [6,16,25,32,33]; the type of exercise they should perform are: get up and sit from a chair, go down and climb stairs, squats [16,25] and plyometric exercises [6], as these help prevent sarcopenia, decrease bone density, prevent falls and hip fractures [48]. It is also important because strength helps us to value the functional capacity of the person. These benefits are obtained by lifting weights, rubber bands, or using bodyweight, these being the most recommended [16,25,33]. The frequency should be 2–3 days per week [25,32] with a volume of 3 series of 12 repetitions [16]. This pattern was included in the range of repetitions established by the studies of Cervera and Villanueva & Fernández [48,49] who suggest –15 minimum repetitions to obtain benefits.

Papers that have recommended strength training exercises in children [6,7,14] suggest activities such as callisthenic activity, jumps, and high-impact activities such as badminton or tennis [14]. These activities at an early age improve strength, potency, speed, and performance in motor skills, as well as improving bone health, increasing density and bone mineral content. It may also be interesting to perform plyometric exercises even if this has not been recommended [52]. The recommended frequency is 3 days per week using bodyweight or with light weights, according to Peña et al. [52].

### 4.3. Recommendations for Flexibility-Stretching and Relaxation-Meditation

Based on the flexibility and relaxation guidelines the articles that are aimed at the general population recommend exercises for flexibility and stretching [7,12,16,18,19,20,29,31,33] and yoga or Pilates [10,12,13,16,17,28,30]. Previous studies [53,54] showed that these exercises improve mobility of joints and present benefits in the tendon units of the muscles, increasing strength and endurance. These benefits are obtained with a 10-min flexible routine; Faigebaum [55] shows that flexibility has benefits or impacts on health, daily life, and athletic performance, as it decreases the risk of injury and muscle stiffness, increases muscle relaxation and mobility and improves neuromuscular coordination. In the recommendations or guidelines, workload of 20″–30″ or 30″ [7,29] is recommended to improve flexibility [53]. Although we do not know the weekly frequency recommendations, Fernando [43] recommends between 5–7 days a week dynamic and static stretches so as not to lose joint mobility range and improve flexibility, on at least 2–3 days weekly. These recommendations also apply to patients [9].

Yoga is a highly recommended activity in the general population [7,10,19,20,28]. Defined as a combination of relaxation, meditation and breathing [56,57], it has benefits that can help in the COVID-19 situation, as it helps calm stress, acts on the immune system, and improves health. Other additional benefits include improved memory, concentration, and self-efficacy [56,57].

Flexibility in the over 65s is also recommended [16,33] by stretching or mobility, bending and extension exercises [33] The volume recommended is 15″–30″ or 3 series of 3″–10″ [16,33]. Flexibility in older adults helps increase the range of motion of the worked joints [48,58]. To be effective, both dynamic and static stretches must be performed between 10”–30”.

### 4.4. Recommendations for Balancing Activity

As for this type of work, the elderly must perform balancing activity and fall prevention [29,32] Activities were pro-set such as walking a line, tiptoe walking, or using unstable elements [25]. In the case of this population, it is recommended to perform this type of activities for 2 or 3 sessions per week Previous studies show that exercising balance helps to maintain the control of the body, avoiding falls [48,59]. El Taichi helps to improve balance in majors and reduces the risk of falls, in addition to finding benefits in other physical or psychic aspects and helping active aging. To obtain these benefits exercise can be carried out for 2–3 sessions as recommended in this review [60,61].

As a summary of discussion and all the recommendations found according to the type of population:

For children, volumes of aerobic exercise recommended are 60 min/day for those over 5 years old and 180 min per day for younger achieving a total of 300 min per week. As for strength training, the recommended frequency is 3 days per week.

For adults and older people, the most of recommended weekly frequency is one session per day of aerobic exercise with volumes of between 30–60 min sessions. As for strength training, the recommended frequency is 3 days per week. As for flexibility training, the benefits are obtained with a 10-min flexibility routine, and as for balance training, it is recommended to perform this type of activity for 2 or 3 sessions per week.

For adults with diseases, the frequency of aerobic exercise has been recommended as 5–7 days per week and a volume of 150 min per week or 30 min per session. As for strength training, the frequency proposed was 2–3 days/week.

Finally, we would like to present some recommendations for maintaining physical activity for individuals following different levels of restriction [62]. Thus, all the types of exercises and recommendations grouped in this study can be performed by each subject in his own home, but without having to keep a social distance if they live alone or if they are people living together in their own household.

For people who use gyms or fitness centers [62], it is recommended that, before going to them, one should use options for online reservations. Furthermore, sone should limit attendance at indoor group training sessions, maintain at least six feet of separation as much as possible, don’t shake hands or touch others, ensure equipment is clean and disinfected, wear a mask when interacting with other people and wash your hands.

For people who visit parks and recreational facilities or playgrounds [62], it is recommended to visit facilities that are close to home; don’t visit crowded parks or campgrounds; carefully consider the use of playgrounds, and help children follow guidelines; stay at least six feet away from people you don’t live with; wear a mask; wash your hands often and don’t share items with people you don’t live with.

To summarize, it should be noted that the recommendations made by the institutions are not fully adapted to the situation and do not take into account the conditions of confinement. With new outbreaks of COVID-19 or new subsequent pandemics, this type of recommendation will be necessary, since up to now they have not been adapted to the different cases of each type of person.

## 5. Limitations and Future Lines of Research

Among the possible limitations, we find a lack of data in some studies, especially about the variables of frequency, volume, intensity and recovery; another limitation is the lack of research studies where recommendations to clarify the guidelines to be followed are implemented. Furthermore, while physical activity promotion is important during these times of confinement, the long-term effects are only postulations. Moreover, this rationale will also depend on the duration of time of the pandemic, which is unknown for now. It is also important to note that the type of restrictions imposed will vary by cohort and country, so this must be taken into account.

Possible future lines of enquiry would be the review or investigation of physical activity recommendations in the new normal, after confinement. Also, recommendations for physical activity could be established for people confined in small households. This review can help the general population and sports professionals to carry out controlled training at home if we have to be confined again because of COVID-19 resurgence. For this reason, this information could help us to be better prepared for the future.

## 6. Conclusions

Exercises are recommended to maintain good physical condition during confinement. However, in the reviewed literature, there are no recommendations to be adapted to all people whatever their situation and disease. Even so, the most recommended guidelines are for aerobic exercise, strength, flexibility-stretching, and balance exercises. The form of exercise prescription from The American College of Sports Medicine (ACSM) (FITT-VP) [63] recommends that all healthy adults should participate in moderate intensity aerobic physical activity for a minimum of 30 min on 5 days per week or vigorous intensity aerobic activity for a minimum of 20 min on 3 days per week. In addition, every adult should perform activities that maintain or increase muscular strength and endurance for a minimum of 2 days per week, without forgetting other components of a healthy lifestyle such as flexibility and balance training [63]. Most of the exercises proposed in this review are common activities in our daily lives and are intended to be carried out at home.

According to these studies, at least 150 min of moderate-intensity physical activity or 75 min of vigorous physical activity per week and 2–3 strength sessions should be performed in children, adults, or older adults. Children should take 60–180 min of daily moderate to vigorous physical activity. In strength training during confinement, it is advisable to use your own bodyweight or light weights to help maintain physical condition.

People over the age of 65 should also exercise with a frequency of 2–3 days per week to avoid risks of falling.

Having reviewed the literature, the vast majority of recommendations for physical activity during the period of the first wave of the COVID-19 pandemic were not actually pandemic-specific. Most recommendations were general and established prior to the pandemic, and future studies need to include specific recommendations for measures taken in each country as a result of the COVID-19 pandemic.

The main contribution of the study is the discovery that the recommendations do not take into account the conditions of home confinement and are not fully adapted. Therefore, it is urged that different institutions create common recommendations that will set the pattern for physical activity at home in times of pandemic.

## Figures and Tables

**Table 1:** Aerobic activity recommendations.

Reference, Year	Population	Type of Activity	Frequency (Days/Week)	Volume (Min/Week)	Intensity	Recovery
American College of Sport Medicine, 2020 [10]	General	Walk	-	150′–300′	Moderate	-
		Dance				
		Static machinery				
		Up and down stairs				
WHO, 2020 [7]	Under 5 years old	Run		1260′		
		Jump	-		-	-
		Catch				
	Children 5–17 years old	Jumping into the rope	-	420′	Moderate and Vigorous	-
		Join aerobic classes				
	Adults	Climbing stairs		75′–150′	Moderate to vigorous	
		Walk				
		Join coves				
		Dance	-			
		Household chores				
		Climb knee to elbow		(Choose 5 sets)		Between each set 30″–60″
		Raising knee to the opposite elbow				
American Heart Association,2020 [11]	General	Scissor jumps		210′ (Choose 3–4 sets of 30”–3 exercises)		
		Squat jumps				
		Run				
		Up and down stairs	-		-	-
		Knee lift				
		Climber				
		Jump				
		Burpees				
		Static machinery				
Chen et al. 2020 [13]	General	Walking at home, going up and down stairs	1 session/day	210′	Moderate	-
			Every 2 days	20 days	Vigorous	-
Fallon, 2020 [18]	General	Dance		150′	Moderate	
		Programs				
		Run	-			-
		Static machinery				
Spanish Society of Sports Medicine, 2020 [33]	People with cardiovascular disease and older	Walk				
		Run				
		Static machinery				
Lippi et al. 2020 [27]	Adults General	Walk		150′–75′	Moderate/vigorous	
		Static machinery				
		Household chores	-			-
		Up and down stairs				
		Step exercises				
Oliveira Neto et al. 2020 [15]	General	March		150′–300′	Moderate RPE (Zone 3/10 or 4/10)	
		Up and down stairs				
		Static machines	-			-
		Jumps (jump to rope)		(30 min/day or 2–5 sets of 10′–15′)		
Guan et al. 2020 [35]	Children 3–4 years old	Active activities	-	1260′	Moderate to vigorous	-
	Children 5–17 years old	-	-	420′		-
Mera et al. 2020 [29]	Children 5–17 years old	-	1	420′	Moderate	-
	General	Walk		150′		-
Song et al. 2020 [34]	Adults with pneumonia	Static machinery	3	90′ (25–30/session)	65–75% Spare Fc	-
Chen et al. 2020 [14]	Children	Bike	1 session/day	420′	Moderate to vigorous	
		Walk				-
		Active games				
Jimenez Pavón et al.2020 [25]	Older	Walk	5–7 days/week	200′–400′	Moderate: 40–60% Reserve Fc o 65–75% Fc max	
		Multi-component exercise				-
		Dance				
Halabchi et al. 2020 [22]	Healthy people	Walk	-		Moderate	-
	Upper Respiratory Tract Infection people limited symptoms	Walk	-	60′	Low-Moderate	-
Hull et al. 2020 [23]	General	-	3–5	90′–300′	60–80% of maximum capacity	-
Ferreira et al. 2020 [19]	Children/teens			300′	Moderate to vigorous	
Hammami et al. 2020 [6]	Children 6–17 years old	Active video games	1	420′	Moderate to vigorous	-


	Active adults	gymnastics, aerobic activities,		150′–75′	Low 50–80% Fcmax, moderate 70–90% Fcmax, high 85–100% Fcmax	
			-			-
	
	Sedentary Adults				Low 50–75% Fcmax, moderate 70–85% Fcmax, high 85–95% Fcmáx	
			-			-
	
	Over 65		-		Low 50–70% Fcmax, moderate 65–80% Fcmax, high 70–90% Fcmax	-
Heart Foundation, 2020 [16]	General	Walking careers				
		Careers to cat				
		Back racing				
		Side jumps with displacements	-	-	-	-
		Walk				
		Dance				
		Aerobic classes				
		Static machinery				
	Children	Circuits around the house	-	-	-	-
	Over 65	Walk		300′		
		Up and down stairs	3/day	(20 steps)	-	-
Eirale et al. 2020 [17]	Athletes	Cycling	3–4	180′–240′ (2 sessions of 30 or 1 session 60)	75–80% Max Fc	
		Swim				-
		HIIT Run				
Ahmed, 2020 [9]	Patients	Walk	5–7	150′	40–59% Vo2máx *	
		Cycling				-
		Cross-trainer				
		Remo				
Jakobsson et al. 2020 [24]	Adults	Walk		150′–75′ 300′–150′	Moderate/vigorous	
		Climbing stairs				
		housework	-			-
		Jogging				
		Jump				
Banerjee et al. 2020 [12]	General	Walk	1	210′ (30′/day or 3 sets/day of 10–15′)	Moderate	
		Jogging				
		Cycling				-
		Treadmill				
		Up and down stairs		(15′/day)		
		Housework				
Nyenhuis et al. 2020 [30]	General	Walk		150′–300′	Moderate	
		Run				
		Virtual classes	-			-
		Bicycle				
Narici et al. 2020 [5]	General	Jump rope		Circuit alternating exercises with high volumes	Baja-moderate	
		Jogging				
		Burpees	-			
		Walk				
		HIIT				
Pitanga et al. 2020 [31]	General	Housework	1	150′–300′	Take the Moderate	
		Up and down stairs				-
	Over 65	Housework	-	150′		-
		Playing with children	-			-
Roschel et al. 2020 [32]	Over 65	-	-	150′–75′ (Rounds of 10′) 300′ 150′	Moderate/vigorous	-
Maycon Junior Ferreira et al. 2020 [20]	Adults	Tasks of daily life	-	150′	-	-
	Children	Jump	-	300′	-	-
		Energy-spending games				
Jukic et al. 2020 [26]	Athletes	Static machinery	-	-	-	-
		Circuit exercises	-	-	-	-

* Vo2max: Maximum oxygen consumption, Fc max: maximum heart rate, RPE: perceived stress scale.

**Table 2:** Strength training recommendations.

Reference, Year	Population	Type of Activity	Frequency (Day/Week)	Volume (Min/Week)	Intensity	Recovery
American College of Sport Medicine, 2020 [10]	General	Squats	2			
		Sit-up from the chair				
		Pushups		-	-	-
		Lunges				
		Lunges/rises to one leg				
WHO, 2020 [7]	Children 5–17 years old		3	-	Lifting weights	-
	Adults	Pushups	2		Corporal weight or weights	
		Squats		5 (10–15)		30″–60″
		Plates		20″–30″		
		Back extension		5 (10–15)		20″–30″
		Quadrupeds with two supports		5 (20–30)		30″–60″
		Buttock bridge		5 (10–15)		30″–60″
		Triceps backgrounds		5 (10–15)		30″–60″
American Heart Association, 2020 [11]	General	Plates		Choose 3–4 30″–3″ exercises		
		Pushups				
		Abdominal				
		Hip elevation	-		-	-
		Triceps backgrounds				
		Lunges and squats				
Spanish Society of Sports Medicine, 2020 [33]	People with cardiovascular disease and older	Arm			Lightweight: body weight or lifting weights (250 g–500 g)	
		Shoulder				
		Back	-	-		-
		Legs				
		Hips				
	General	Strength exercise	-	-	Body weight, weights, elastic bands	-
Chen et al. 2020 [13]	General	Thrusts			Lifting or carrying weights	
		Get up and sit down				
		Squats	-	-		-
		Abdominal				
		Pushups				
Fallon, 2020 [18]	General	Stairs	2	Perform many repetitions	Lifting low weights % of load	
		Squats				
		Pushups				-
		Lifting legs on ladders				
Ahmed, 2020 [9]	Patients	Calisthenics	2–3	2–4 × 8–12	Body weight/bands/weights; Gradual increase 40–50% to 60–70% 1RM	
		Machine exercises				-
Jiménez Pavón et al. 2020 [25]	Over 65	Jumps				
		Squats	2–3 ≥2		Corpora weight or light and moderate weights	
		Sit-up from the chair		-		-
		Up and down stairs				
Lippi et al. 2020 [27]	Adults	Exercises with elastic bands			Belt strength and material weight	
		Exercises with balls		-		-
		Weight exercises				
Heart Foundation, 2020 [16]	General	-	-		Weights, 1kg food packs, elastic bands	-
	Over 65	Get up and sit in a chair				
		Force in hands with a towel: make it look like you are manually wringing		3 × 12	Body weight	-
Eirale et al. 2020 [17]	Athlete	Pushups	2	60/session at most	Not use loads max; limited to 80% FC max	
		Squats				-
		Abdominal				
Jakobsson et al. 2020 [24]	General	Squats		5 × 10–15		1′
		Bridges	-		-	
		Back extension				
		Jumps				
		Plates				
Mera et al. 2020 [29]	General	Squats	-			
		Pushups		-		-
		Abdominal				
Mattioli et al. 2020 Mattioli [28]	General	Get up and sit in a chair	-	-	Lightweights	
		Up and down stairs				
Nyenhuis et al. 2020 [30]	General	Squats		Sets 3–5 do the 5 exercises	Body weight/Elastic bands or light weights	
		Mountain climber				
		Burpees				
		Pushups				
		Abdominal				
Pitanga et al. 2020 [31]	General	Squats				
		Pushups	-	-	-	-
		Abdominal				
		Up and down stairs				
Hammami et al. 2020 [6]	Children		3	-	Exercise with body weight	-
	Adults	Pushups	2			
		Box jumps				
		Burpees		-		
		Rope jumps				
		Squats		Beginner: 1–2 × 10		
				Advance: 2–3 × 20		
		Lizards		Beginner: 1–2 × 10		
				Advance: 2–3 × 20		
		Thrusts		Beginner: 1–2 × 5		
				Advance: 2–3 × 10		
		Scissor jumps		Beginner: 1–2 15		
				Advance: 2–4 × 20		
		Abdominal		Beginner: 1–2 × 15		
				Advance: 2–4 × 20		
		Planks		Beginner: 1–2 × 20”		
				Advance: 2–4 × 40″		
	Over 65	Plyometric exercises		-	-	-
		resistance				
Narici et al. 2020 [5]	General	-	-		Charge at 50% 1RM, 3″ in concentric motion and 3″ eccentric movement	-
				High Reps (24)	Low loads 30% 1RM	
Roschel et al. 2020 [32]	Over 65	Exercises for large muscle groups	≥2			
Jukic et al. 2020 [26]	Athletes	Vertical jump			Elastics bands/weights//medicine balls	
		Jump horizontal				
		Plyometric				
		Eccentric exercises	-	-		-
Oliveira Neto et al. 2020 [15]	General	Plates	2–3	1–4 × 8–20	Manual resistance or self-resistance	
		Side Bridge				
		Bird-dog				
		Pushups				
		Suspended/tower row				-
		Shoulder push up				
		Squats				
		Lunges				
		Good morning				
		Unilateral calf raises				

**Table 3:** Recommendations for flexibility-stretching, relaxation-meditation.

Reference, Year	Population	Type of Activity	Frequency (Day per Week)	Volume (Set × Repetitions or Seconds)	Intensity
American College of Sport Medicine, 2020 [10]	General	Yoga			
		Mindfulness	-	-	-
WHO, 2020 [7]	General	Balance postures			
		Stretching	-	20″–30″	-
		Meditation		5′–10′	
Spanish Society of Sports Medicine, 2020 [33]	People with cardiovascular disease and older	Stretching		15″–30″	
		Mobility exercises			
		Bending and extension exercises	-		-
		Joints: Knee, ankle, wrist, hip, shoulder and elbow			
	General	Flexibility exercises	-	-	-
Fallon,2020 [18]	General	Stretching			
		Yoga	-	-	-
		Pilates			
Maycon Junior Ferreira et al. 2020 [20]	General	Stretching			
		Relaxation	-	-	-
		Meditation			
Heart Foundation, 2020 [16]	General	Stretches: upper and lower train,	Every 1:30 am or 2 h	-	-
		yoga			
	Older	Stretching arms	-	3 × 3 of 10″	-
Eirale et al.2020 [17]	Athletes	Dynamic stretches	2–3		-
		Static stretches			
Ahmed, 2020 [9]	Patients	Static	2–3	2–4 × 10″–30″	Feeling slight oppression
		Dynamic			
		Pilates			
		Yoga			
Mera et al. 2020 [29]	General	Joint mobility	-	-	-
		Dynamic stretches		30″	
Mattioli et al. 2020 [28]	General	Relaxation			
		Deep breaths	-	-	-
		Yoga			
Banerjee et al.2020 [12]	General	Yoga			
		Stretching	-	-	-
Chen et al. 2020 [13]	General	Yoga	-	-	-
M.J. Ferreira et al. 2020 [19]	General	Relaxation			
		Meditation	-	-	-
		Stretching			
Nyenhuis et al. 2020 [30]	General	Yoga	-	-	-
Pitanga et al. 2020 [31]	General	Stretching	-	-	-

**Table 4:** Balance recommendations.

Reference, Year	Population	Type of Activity	Frequency (Days/Week)
Chen et al. 2020 [13]	General	Tai Ji Quan	-
		Qingong	
Jiménez Pavón et al. 2020 [25]	Over 65	Walking on a line on the floor	≥2
		Walking on toes or heels	
		Walking on toes or heels and step on obstacles.	
Heart Foundation, 2020 [16]	General	With a chair, bosu or unstable elements, go down and stand up several times in a row without supporting your forearms	-
Song et al. 2020 [34]	Adults with pneumonia	Tai Ji *	
		Qingong *	-
		Fall prevention exercises	
Mera et al. 2020 [29]	Over 65	Balance	3
Pitanga et al. 2020 [31]	General	Balance	-
Roschel et al. 2020 [32]	Over 65	Fall prevention exercises	3
		Balance	

* Tai Ji and Qingong: exercises that combine meditation, relaxation with movement.
